# The Focal Adhesion Analysis Server: a web tool for analyzing focal adhesion dynamics

**DOI:** 10.12688/f1000research.2-68.v1

**Published:** 2013-03-04

**Authors:** Matthew E Berginski, Shawn M Gomez

**Affiliations:** 1 UNC/NCSU Joint Department of Biomedical Engineering, University of North Carolina at Chapel Hill, Chapel Hill, NC, 27599-7575, USA; 2 UNC Department of Pharmacology, University of North Carolina at Chapel Hill, Chapel Hill, NC, 27599-7575, USA; 3 UNC Department of Computer Science, University of North Carolina at Chapel Hill, Chapel Hill, NC, 27599-7575, USA

## Abstract

The Focal Adhesion Analysis Server (FAAS) is a web-based implementation of a set of computer vision algorithms designed to quantify the behavior of focal adhesions in cells imaged in 2D cultures. The input consists of one or more images of a labeled focal adhesion protein. The outputs of the system include a range of static and dynamic measurements for the adhesions present in each image as well as how these properties change over time. The user is able to adjust several parameters important for proper focal adhesion identification. This system provides a straightforward tool for the global, unbiased assessment of focal adhesion behavior common in optical microscopy studies. The webserver is available at: http://faas.bme.unc.edu/.

## Introduction

The quantitative analysis of focal adhesion (FA) structures in motile cells com­monly relies on the use of fluorescently tagged protein components and time-lapse fluorescence microscopy. Traditionally, the resulting images are analyzed using NIH ImageJ ^1^ or related tools, but we have recently developed a set of computer-vision algorithms designed to automate many of these analysis steps. These core methods have been documented in a prior publication ^2^ and made available as an open source download; however, they require substantial exper­tise with the command line interface for their use. With the focal adhesion analysis server (FAAS), we have created a web application that allows users to submit time-lapse fluorescence image sets of FA proteins and have these images automatically analyzed.

The methods implemented by the analysis system have been previously used in several studies to investigate the quantitative properties of FAs in cells under various conditions. For example, adhesion static and dynamic properties were quantified with fluorescently labeled FAK, vinculin and paxillin ^3– 5^. Global, whole-cell changes to adhesion and cytoskeletal architecture when the Arp 2/3 complex is disabled have also been characterized ^4^. By integrating these image analysis methods into a straightforward web application, we hope to make them more broadly accessible to the cell-imaging community.

## Features

**Figure 1.**
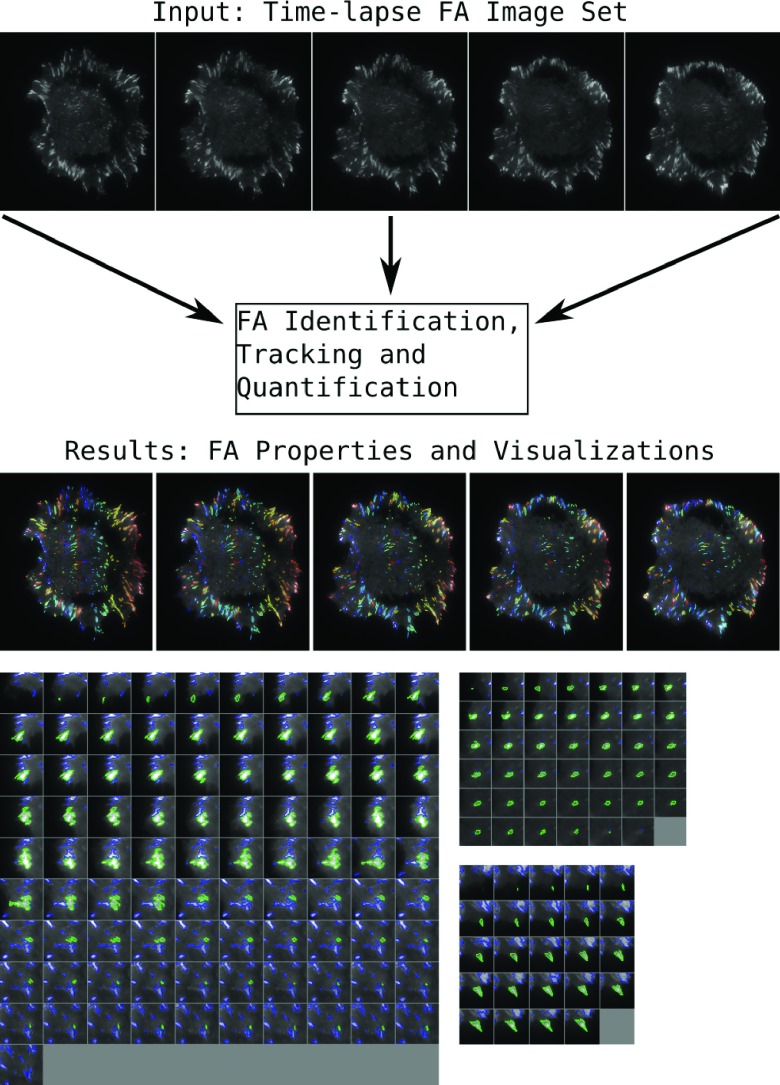
Sample input images and output visualization from the focal adhesion processing pipeline. The results section shows examples from the visualizations produced by the pipeline. In the top example, the entire cell is shown, with an individual adhesion outlined and tracked through time. The bottom three examples show single adhesions, outlined in green, with other nearby adhesions outlined in blue.

The primary interface is a set of webpages that allow a user to upload a stacked tiff set of images for processing. After the images are uploaded to the server, the processing pipeline is run, and the results are returned as a downloadable zip file. This results file contains all the intermediate processing steps as well as a set of visualizations. These visualizations show which regions of the cell were identified as adhesions and how the tracking algorithm followed single adhesions through time ( Figure 1).

### FA properties and visualizations

The analysis pipeline extracts and quantifies a wide range of properties. FA properties characterized in each individual image include adhesion area, marker protein intensity and the lengths of the major and minor axes. In addition to these static properties, the system also collects dynamic adhesion properties, which are quantified by recording the changes in individual adhesions between frames in the image stack. Dynamic properties currently include the FA assem­bly and disassembly rates ^6^ and the focal adhesion alignment index ^4^. All of these results are saved in CSV format, which is suitable for import into statisti­cal or graphing software. For users only interested in static results derived from individual images, as in an analysis of a set of fixed-cell images, all the other dynamic properties can be safely ignored.

The user is also provided with two types of visualizations that show either the entire field of view or single adhesions over time. The visualization of the entire field of view is produced for every image in the submitted image set and outlines each adhesion with a unique color ( Figure 1). This visualization can be used to verify that the adhesions were correctly detected, segmented and tracked. The second visualization type shows a single adhesion segmented and tracked through time ( Figure 1).

Provided that adhesions are present for at least 10 sequential images, this visualization allows the user to compare an individual FA’s properties with the appearance of the adhesion in the original image data. This suite of automatically extracted properties and visualizations enables the user to minimize the amount of laborious manual analysis normally required to quantify FA image sets.

### User adjustable parameters

Several of the parameters used to analyze the images can be specified when an image set is submitted for analysis. The most important of these is the threshold used to identify the regions of the image that qualify as FAs versus background. The appropriate threshold will vary depending on the type of cell imaged and the imaging conditions. To make setting this parameter easier, we have added a feature where a single image can be submitted, segmented using various thresholds and then immediately returned to the user for visual inspection of the results obtained when the threshold is varied. The user also has the option to turn off the default watershed-based segmentation that is used to split adjacent FAs and modify the minimum or maximum FA size accepted by the system. Finally, the time between images can also be specified to ensure that the calculation of the rates of assembly or disassembly are made in the correct units.

Users have the option of providing an email address when an image set is submitted. If an email address is provided, notification of the completion of the image processing pipeline, along with a link to download the results, is sent. The system can also be used without an email address, but the user must return to the web interface to check on the status of the processing. The processing time is dependent on the number of images in the set and how many adhesions are detected during processing. Using typical input data, we tested the system throughput and found that the average processing and analysis time per image, under full load, is 13 seconds. Because the system can handle four image sets at once, we expect experimental throughput to be acceptable for everyday usage.

## Conclusion

The Focal Adhesion Analysis Server provides an automated image processing pipeline in an easy-to-use web-based application. A wide range of FA properties are automatically collected from the image sets submitted, and the results are returned in CSV formatted files. Users have the option to adjust the parameters used to process their image sets to suit their specific imaging conditions and cell types of interest.
