# Supplementary material for: The Focal Adhesion Analysis Server: a web tool for analyzing focal adhesion dynamics
Source: F1000Res. 2013 Mar 4;2:68. [Version 1] doi: 10.12688/f1000research.2-68.v1 (PMC3752736; doi:10.12688/f1000research.2-68.v1)
Supplement: Peer review history

# Peer review: Referee response for version 1

**Published:** 2013-03-07

**Authors:** Kenneth Yamada

**Affiliations:** Laboratory of Cell & Developmental Biology, National Institute of Dental and Craniofacial Research, National Institutes of Health, Bethesda, MD, USA

This automated web-based analysis system provides a helpful system for considerably simplifying analyses of dynamic focal adhesions of cells in 2D cell culture. Although two examples of the analysis of single adhesions are clear, the example at the bottom left of Figure 1 seems to show appearance of a rather large, complex, multi-pronged cell adhesion that may be a composite of several smaller focal adhesions – it may be useful to discuss this conceptual issue briefly. After further validation and use by other colleagues, this analysis server may become a valuable shared resource for the field.

I have read this submission. I believe that I have an appropriate level of expertise to confirm that it is of an acceptable scientific standard.

# Peer review: Referee response for version 1

**Published:** 2013-03-05

**Authors:** Jonathan Jones

**Affiliations:** Department of Cell and Molecular Biology, Northwestern University Medical School, Chicago, IL, USA

The authors have generated a valuable tool for analysing adhesion site dynamics. The article is clear, logical and focused. This should be a useful resource for the field.

I have read this submission. I believe that I have an appropriate level of expertise to confirm that it is of an acceptable scientific standard.
